# Comparative Analysis of Microbial Community Structure and Function in the Gut of Wild and Captive Amur Tiger

**DOI:** 10.3389/fmicb.2020.01665

**Published:** 2020-07-24

**Authors:** Yao Ning, Jinzhe Qi, Michael T. Dobbins, Xin Liang, Jingxuan Wang, Shiyu Chen, Jianzhang Ma, Guangshun Jiang

**Affiliations:** ^1^College of Wildlife and Protected Area, Northeast Forestry University, Harbin, China; ^2^School of Forestry, Northeast Forestry University, Harbin, China; ^3^Department of Wildlife, Fish, and Conservation, University of California, Davis, Davis, CA, United States

**Keywords:** Amur tiger, 16S ribosomal RNA gene sequencing, core microbiome, metagenome sequencing, conservation management

## Abstract

It has been well acknowledged that the gut microbiome is important for host health, composition changes in these microbial communities might increase susceptibility to infections and reduce adaptability to environment. Reintroduction, as an effective strategy for wild population recovery and genetic diversity maintenance for endangered populations, usually takes captive populations as rewilding resource. While, little is known about the compositional and functional differences of gut microbiota between captive and wild populations, especially for large carnivores, like Amur tiger. In this study, high throughput sequencing of the 16S ribosomal RNA (rRNA) gene (amplicon sequencing) and metagenomics were used to analyze the composition and function variations of gut microbiota communities between captive and wild Amur tiger populations based on total 35 fecal samples (13 from captive tigers and 22 from wild tigers). Our results showed that captive Amur tigers have higher alpha diversity in gut microbiota, but that the average unweighted UniFrac distance of bacterial taxa among wild Amur tigers was much larger. The function differences involve most aspects of the body functions, especially for metabolism, environmental information processing, cellular processes, and organismal systems. It was indicated that the diet habit and environment difference between captive and wild populations lead to composition differences of gut microbiota and then resulted in significant differences in functions. These contrasts of functional and compositional variations in gut microbiota between wild and captive Amur tigers are essential insights for guiding conservation management and policy decision-making, and call for more attention on the influence of gut microbiota on the ability of captive animals to survive in the wild.

## Introduction

The gastrointestinal tracts of vertebrates are inhabited by large and diverse populations of bacteria, which play an integral role in food decomposition, nutrient supply, immune modulation, pathogen prevention, and may also be an essential factor in influencing the processes of ecological adaptation (Doolittle, 1998; Round and Mazmanian, 2009; Turnbaugh et al., 2009; Honda and Littman, 2012; Alberdi et al., 2016). Gut microbiota are also indispensable for maintaining the health of hosts, because microbial imbalances may result in changes in the host’s microbial diversity and community composition, potentially leading to inflammatory bowel disease, infectious diseases, obesity, and autoimmune disorders (Frank et al., 2007; Turnbaugh et al., 2009; Moeller et al., 2014; Brune and Dietrich, 2015; Clayton et al., 2016). In return, these host-microbial communities are potentially shaped by intrinsic host traits and extrinsic environmental factors. Host genetics, stomach PH, and antimicrobial peptides, as the intrinsic host traits, could modify the gut microbial community through imposing selection filters (Xia et al., 2014). Among the external factors, diet and surrounding environmental factors are major drivers which substantially influence microbial community composition (Xia et al., 2014). The increasing knowledge of gut microbiota is mainly derived from studies on animals that are germ-free and laboratory-based, which has limited application to animals in the wild (Kohl and Dearing, 2014; Kreisinger et al., 2014). Characterizing the gut microbiota of mammals, living under natural conditions, is an important health issue and has significant impacts on understanding the ecological and evolutionary relationship between hosts and their gut microbiota (Nelson et al., 2013).

Amur tiger (*Panthera tigris altaica*) represents a charismatic flagship species of the boreal forest of Asia and once was widely distributed across Northeastern China, the Korean Peninsula, and Russian Far East (Ning et al., 2019). However, the wild population dropped sharply in the late 19th century, due to habitat fragmentation, indiscriminate poaching, and other caused by intensified human disturbance, leading it to be highly endangered throughout its range (Wang et al., 2018). The Chinese government has shown great effort to protect this endangered big cat, especially after the leaders of 13 countries reached the consensus on doubling the wild tiger population at St. Petersburg Tiger Forum in 2010 (Kilian, 2010). For instance, the central government of China initiated the Northeast Tiger Leopard National Park in 2015, which covered 14,600 km^2^ area of the most important Amur tiger habitat in China (Li et al., 2016). And in 2019, the National Forestry and Grassland Administration of China hosted the first International Forum on Tiger and Leopard Transboundary Conservation in Harbin, which attracted more than 300 representatives from 19 regional countries and substantially promoted the international cooperation on tiger and leopard conservation^1^. Although, the tiger population in China has increased significantly recently under strict conservation regulations, most tiger individuals are still only distributed along the Sino-Russian border (Dou et al., 2016; Wu et al., 2016; Wang et al., 2018). Their inward spread into China was seriously limited by many human disturbance factors and a lack of prey (Wu et al., 2016). In the current situation, reintroduction may be a better choice for maintaining and quickly restoring tiger populations across multiple habitat patches within Northeast China, given that Russia has re-populated Amur tigers to Jewish Autonomous Oblast’s Forest successfully through reintroduction. What is more, the Siberian Tiger Park in Heilongjiang province of China, the largest captive breeding center for Amur tiger in the world keeps more than 1,400 individuals and could provide sufficient resources for reintroducing Amur tigers into the wild in China (http://www.dongbeihulinyuan.com/index.php, in Chinese).

Wild Amur tigers survive in many vegetation types, including deciduous forests, coniferous forests, and natural shrub lands. Their home-range size can often be up to 390 km^2^ and predominantly prey on native medium and large ungulates, such as red deer (*Cervus elaphus*), wild boar (*Sus scrofa*), and roe deer (*Capreolus pygargus*; Jiang et al., 2014). While, captive individuals live in an environment greatly different from wild populations, both in diet and environment, and they are confined to a very limited area and fed duck, chicken, and beef daily. Even so, many relevant studies suggested that captive Amur tiger still retain the abilities of native prey recognition and has similar extent and distribution of genetic variation found within wild population (Henry et al., 2009; Wang et al., 2019). However, it is still unknown whether these substantial diet and environmental differences between captive and wild tigers would result in variations within gut microbiota, and how they may alter gut microbiota functionality.

In this study, we conducted an analysis of the fecal microbial diversity and function in both wild and captive Amur tiger populations to investigate (1) the gut microbiota compositional variation between captive and wild tigers, (2) the core microbiota in Amur tigers, (3) the gut microbiota’s functional differences between captive and wild tigers, and (4) how the gut microbiota composition of the Amur tiger is related to its the functions? To guide the reintroduction of captive individuals to wild, our research systematically and comprehensively investigates the variations of gut microbiota between captive and wild Amur tigers to provide valuable references for further understanding co-development and co-evolution of gut microbiota between captive and wild large carnivores and to also provide important management considerations for the reintroduction of captive Amur tigers to the wild.

## Materials and Methods

### Sampled Materials

Fecal samples of Amur tigers were collected from both captive and wild individuals. Wild samples were predominantly collected through tracking Amur tiger footprints in the snow and encouraging local forestry workers to collect samples while on patrol. We established a standard procedure for sample collection and storage, since the quality of field samples could be affected greatly by the duration left in the field. Most of the field samples were collected in winter when the field temperature could be lower than −20°C. Fecal samples in the field were first identified to species by amplifying the cytochrome b sequence, and then we uncovered individuals by utilizing eight microsatellite loci (Sugimoto et al., 2006; Ning et al., 2019). We used only the best quality samples of each individual for gut microbiota analysis.

Fecal samples of captive tigers were collected in Siberian Tiger Park, China, during the winter, and healthy Amur tigers were randomly selected. Fecal samples were collected immediately after defecation and stored at −80°C for subsequent extraction of DNA after transportation to the laboratory. All selected captive tigers were not given antibiotics or other medicines for 3 months before this study.

### 16S rRNA Gene Sequencing

We extracted microbial community genomes from frozen fecal samples of 22 wild individuals and 13 captive individuals using the E.Z.N.A.®Stool DNA Kit (D4015, Omega, Inc., USA) according to the manufacturer’s protocol. Total genomic DNA was subjected to PCR amplification targeting a 400 bp fragment encompassing the V3 and V4 hypervariable of the 16S rRNA gene using the universal bacteria primer set 338F (5'-ACTCCTACGGGAGGCAGCAG-3') and 806R (5'-GGACTACHVGGGTWTCTAAT-3') and was slightly modified to mitigate the issues caused by low sequence diversity amplicons (Fadrosh et al., 2014). PCR-reactions contained 12.5 μl of pusion hot start flex 2X Master Mix, 2.5 μl of each primer, 50 ng sample DNA, and 25 μl of DNA-free water. Negative control reactions contained all components but ultrapure water replaced the sample solution throughout the PCR amplification to eliminate the possibility of false PCR results. PCR were carried out at an initial denaturing temperature of 98°C for 30 s; 35 cycles of denaturation at 98°C for 10 s, annealing at 54°C for 30 s, extension at 72°C for 45 s, and then final extension at 72°C for 10 min. The PCR products were confirmed with 2% agarose gel electrophoresis, and then purified by AMPure XT beads (Beckman Coulter Genomics, Danvers, MA, USA). The purified amplicons were quantified by Qubit (Invitrogen, USA) and pooled in equal concentrations and sequenced using an Illumina MiSeq platform according to the manufacturer’s recommendations.

Paired-end reads were assigned to samples based on their unique barcode and truncated by cutting off the barcode and primer sequence and merged using FLASH. Preprocessed sequences were clustered at 97% nucleotide sequence similarity level by Vsearch (v. 2.3.4; Rognes et al., 2016) and the representative sequences were chosen for each operational taxonomic unit (OTU) by picking the most abundant sequence within each OTU. Taxonomic data were then assigned to each representative sequence using the Ribosomal Database Project (RDP) classifier and used the Mann-Whitney U test to assess the significant differences between wild and captive tigers.

OTUs abundance information was normalized using a standard of sequence number corresponding to the sample with the least sequences. Alpha diversity is applied in analyzing complexity of species diversity through four indices, including Chao1, Shannon, Simpson, and Observed species, which were calculated using QIIME (v. 1.8.0) software (Caporaso et al., 2010). These indices were compared with Kruskal-Wallis test.

Unweighted UniFrac distances account for unique OTUs and weighted UniFrac distances include information of taxonomic abundance. Both are used to assess differences in gut microbiota communities between individuals, and the analysis of similarities (ANOSIM) was used to test the differences between the two groups. In addition, we calculated the average distance by using the UniFrac’s analysis between every pair of guts to measure the similarity of total gut microbiota in different individuals of the same group (Xu et al., 2017), and its values range from zero to one, where two guts having the same communities is given a value of zero. Principle co-ordinates analysis (PCoA) was implemented to visualize natural groupings of the samples using the vegan package in R software, based on weighted and unweighted UniFrac distances.

### Core Microbiome

As the core microbiome may be critical to the overall function of those communities, we further analyzed the core microbiome of Amur tigers. We applied the composition method, which is based on the relative abundance of OTU to identify the core OTU firstly by using Metagenomics Core Microbiome Exploration Tool (MetaCoMET; Wang et al., 2016). To analyze the differences in core microbiome between captive and wild populations, the *t* test in the STAMP software was used to check for significant differences in genus levels between the sample groups. Then, we used the network analysis to visualize the interaction and importance of the microbiome at phylum level by limiting the threshold of *p* < 0.01 and *R* > 0.8.

### Metagenome Sequencing and Analysis

To further explore the functions variation, we filtrated six wild and six captive samples belonging different individuals for metagenomic sequencing analysis on Illumina sequencing HiSeq platform. We used Trimmomatic v. 0.39 to trimm and split sequencing reads into paired and unpaired categories (Bolger et al., 2014) and used bowtie 2 to remove the host genome^2^ (Bolger et al., 2014). Then, the short Illumina reads were assembled into contigs according to the default parameters in MEGAHIT v. 1.1.3 (Li et al., 2016). Individual genomes were annotated by using Prokka v. 1.13.3 (Torsten, 2014) and the accurate computational methods were applied for the quantification of gene abundances in salmon v. 0.14.0 (Patro et al., 2017). The determination of the taxonomic profiles was realized by matching the contigs to the NCBI database and evolutionary genealogy of genes in Kraken2 v. 2.0.8 (Wood and Salzberg, 2014). Nonsupervised orthologous groups (EggNOG) database was searched for the functional annotation and assigning KEGG orthology (KO) numbers to each gene. To identify potential sequences belonging to known carbohydrate-active enzymes (CAZy) families, dbCAN2 web server was performed for automated CAZyme annotation (Zhang et al., 2018). Abundant different features of KEGG Level2 and CAZyme were determined using linear discriminant analysis (LDA) effect size (LEfSe) and setting 3 as the threshold on the logarithmic LDA score (Segata et al., 2011).

To assess accurate overall relationship between the gut microbiota and functions, only the relative abundance of taxonomy ID > 0.1% at least in one sample was considered in the cluster analysis to avoid artificial associations. Taxonomy ID and KEGG Level2 were clustered by using hierarchical agglomerative clustering and clustering by function, respectively. The taxonomy ID cluster was used with the hclust function in R software and Euclidean distances were root sum-of-squares of differences for calculating dissimilarities between observations, while the agglomeration method was used in five different methods, including single, complete, average, centroid, and ward.D. The best cluster method was picked according to the cluster structure, and the silhouette plot was performed to help select the proper number of clusters according to the range of average silhouette width. Spearman method was used to analyze the correlation.

## Results

**Figure 1:**
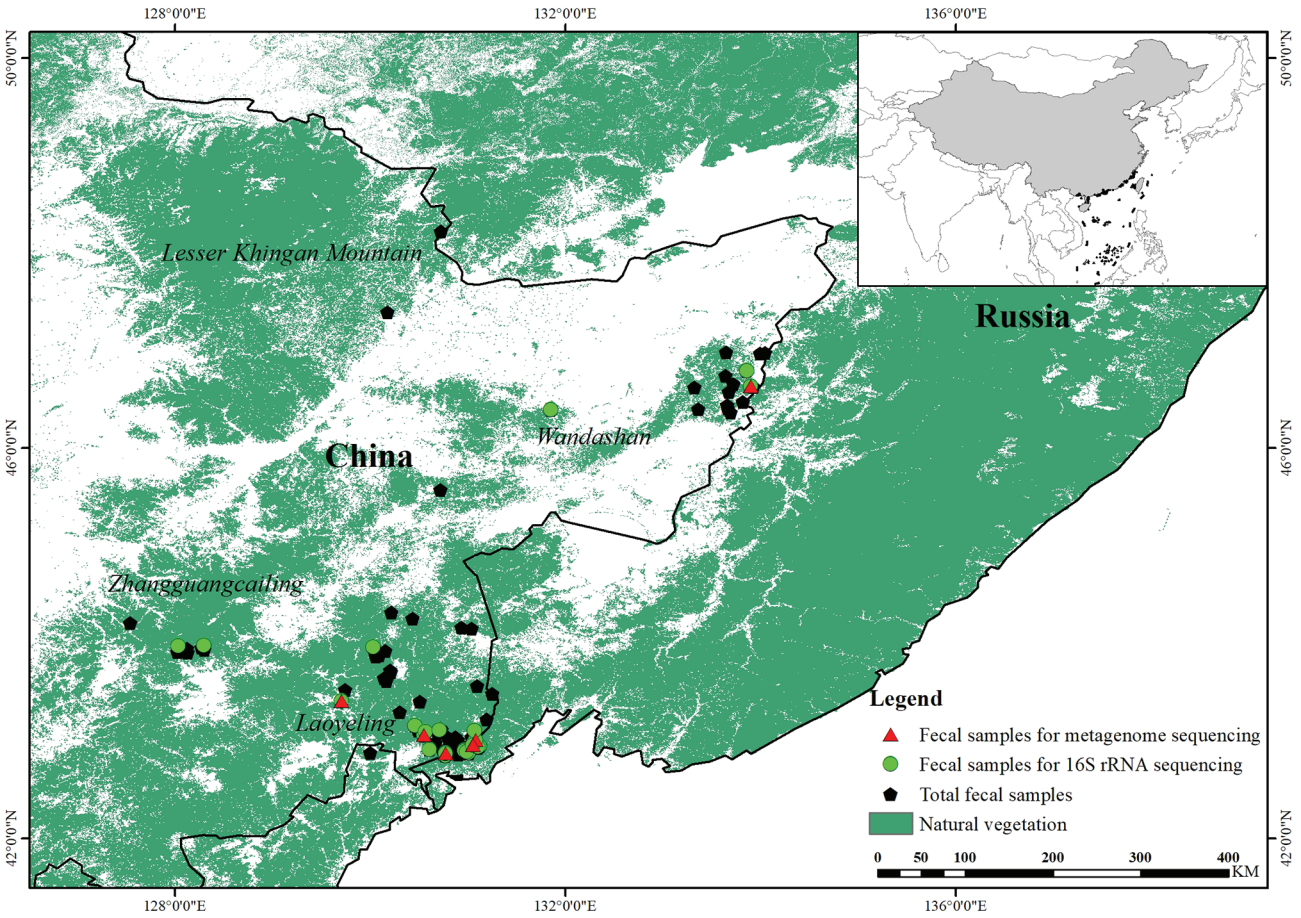
Diagram of sample collection of wild Amur tiger (150) and 16S ribosomal RNA (rRNA) gene sequencing (22) and shotgun metagenome sequencing (12).

Through species identification and microsatellite genetic markers, 150 of 163 fecal samples collected from the wild were confirmed to be tigers and a total of 30 individuals were identified. Twenty-two fecal samples with optimal quality from different individuals were chosen for further analysis (Figure 1). In addition, we collected fresh fecal samples from 13 healthy tigers in Siberian Tiger Park to analyze the gut microbiota composition and function of captive tigers.

### 16S rRNA Sequencing Description

We performed amplicon sequencing of the V3 and V4 region of 16S ribosomal RNA (rRNA) gene on fecal samples collected from captive and wild tigers. After merging the paired-end reads, quality filtering on raw tags and removal of chimeric sequences, we recovered 797,191 reads and the number of sequences per individual sample ranged between 9,732 and 52,870. Amur tiger fecal samples were successfully sequenced with an average of 22,776 reads per individual. These reads were assigned to 1,186 unique phylotypes (OTUs) and 99.07% were successfully classified at the phylum level (*n* = 17), 98.73% to class (*n* = 35), 98.39% to order (*n* = 59), 97.38% to family (*n* = 125), and 88.61% to genus (*n* = 260) using the RDP. The major phylum of Firmicutes, Proteobacteria, Actinobacteria, and Bacteroidetes were contributed to 93.95% of the total microbiome abundance. At the genus level, *Collinsella* up to 17.94% of gut microbiome in Amur tiger, and the other three dominant genera were *Clostridium_sensu_stricto*, *Blautia*, and *Sphingomonas*.

### Composition Differences in Gut Microbiota Between Captive and Wild Amur Tiger Populations

**Figure 2:**
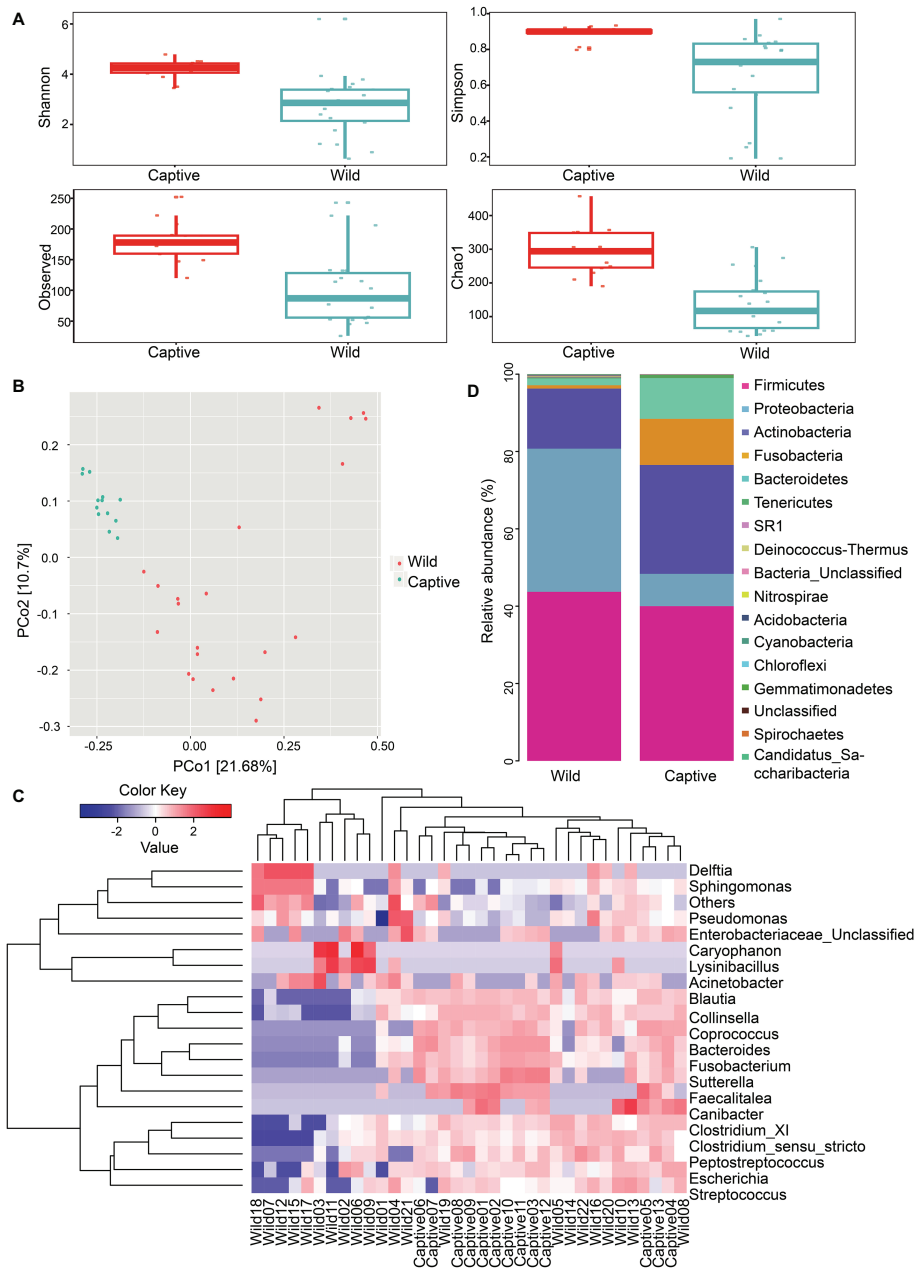
Gut microbiota and functional characteristics were different between wild and captive tigers. The box plot showing the Chao1 and Observed index of alpha diversity between wild and captive Amur tiger **(A)**. Shannon and Simpson index of alpha diversity indicates the differences between wild and captive Amur tiger **(B)**. Principal coordinates plot between all samples were generated with unweight UniFrac distance **(C)**. The top 20 genera bacteria were selected for heat map analysis based on the species richness and the color change to reflect the variation in species abundance **(D)**.

Sequencing analysis indicated that species richness of gut microbiota was significantly different between wild and captive tigers (Chao1: wild, 138.41 ± 78.93; captive, 292.79 ± 73.56; *p* < 0.001) and other indexes showed similar results, such as Shannon (wild, 2.77 ± 1.22; captive, 4.19 ± 0.39; *p* < 0.001), Simpson (wild, 0.66 ± 0.24; captive, 0.89 ± 0.04; *p* < 0.001), and Observed species (wild, 102.77 ± 58.58; captive, 182 ± 34.33; *p* < 0.001; Figure 2A). We used unweighted UniFrac distances throughout the analysis because they provided better clustering to separate the wild and captive group than weighted UniFrac distance. Results of clustering were most likely due to the presence or absence of key taxa in different groups rather than changes in the proportion of dominant members of the microbiota. The non-parametric ANOSIM detected that the inter-group differences between wild and captive ones were significantly greater than the intra-group differences in community composition and abundance (*r* = 0.33, *p* = 0.001). By measuring the fraction of branch lengths in the phylogenetic tree, we calculated the average distance within the community. The results showed that the mean value of unweighted UniFrac distance was 0.717 in wild tigers and was 0.551 in captive tigers. The unweighted UniFrac distances through PCoA revealed that the first PCo axis accounted for 21.68% of the total variability detected in the resemblance matrix (Figure 2B). We classified our OTUs to the level of genus, and found that the gut microbiota of wild tigers were dominated by *Sphingomonas*, *Collinsella*, *Clostridium_sensu_stricto*, and *Lysinibacillus*, while the most abundant genera for captive tigers were *Collinsella*, *Blautia*, *Fusobacterium*, and *Bacteroides*. Total 76 genera were significantly different in abundance between wild and captive tigers, for instance, the level of gram-positive *Lysinibacillus* and gram-negative *Sphingomonas* were much higher in the wild tigers (some of them were showed in Figure 2C). The gut bacterial communities differed in relative abundance between wild and captive tigers also shows in other categories, total four phyla, 29 families were significantly different based on the Mann-Whitney U analysis. The four most dominant phyla, which significantly different between wild and captive tigers, were Actinobacteria ($\bar{X}$ = 15.54 ± 21.56%; $\bar{X}$ = 28.16 ± 23.02%), Fusobacteria ($\bar{X}$ = 0.86 ± 1.9%; $\bar{X}$ = 11.92 ± 12.95%), and Bacteroidetes ($\bar{X}$ = 1.77 ± 3.45%; $\bar{X}$ = 10.54 ± 12.59%; Figure 2D).

### Determination of the Core Bacterial Microbiome

**Figure 3:**
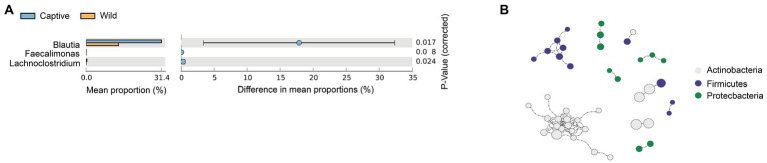
*t* test shows a significant of genus between the wild and captive Amur tiger, the colors were highlighted in green **(A)**. Spearman correlation networks analyze the co-occurrence patterns between phyla of core microbiota and the size of each node was the proportional of community abundance **(B)**.

To analyze the structural basis of metacommunities for Amur tiger, we identified the core microbiome. A total of 109 core OTUs, which account for 21.46% of the total abundance of the gut microbiota, were identified. Network analysis focusing the potential interactions among the core bacterial phyla recognized a total of 79 positive correlations and 10 modules among three different phyla. The differences in core microbiome showed that, in genus level, the relative abundance of *Blautia*, *Faecalimonas*, and *Lachnoclostridium* were significantly higher in captive Amur tiger than the wild population, especially for *Blautia*, the relative abundance in captive Amur tigers was 1.6 times of wild tigers (Figure 3A). The network captured a large part of the correlations, where the interactions within each phylum and two modularity revealed that Firmicutes were positively related to Actinobacteria (Figure 3B).

### Function Differences in Gut Microbiota Between Captive and Wild Amur Tiger Populations

**Figure 4:**
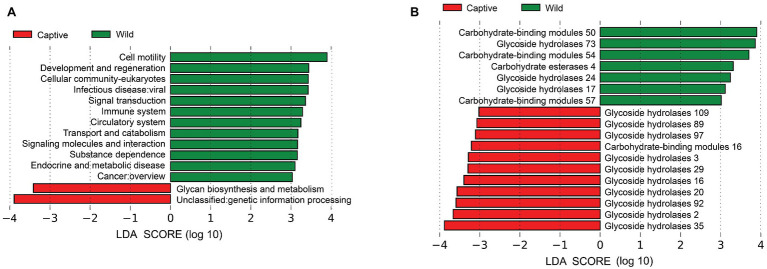
Linear discriminant analysis (LDA) effect size (LEfSe) analysis of KEGG Level2 **(A)** and CAZy **(B)** between wild and captive tiger.

Metagenomic analysis confirmed 7,711 KOs, including 50 KEGG Level2 categories. Wild tigers displayed high abundances in KEGG Level2 categories of cell motility, development and regeneration, cellular community-eukaryotes, and infectious disease: viral, signal transduction, immune system, circulatory system, transport and catabolism, signaling molecules and interaction, substance dependence, endocrine and metabolic disease, and cancer overview. Whereas glycan biosynthesis and metabolism and genetic information processing exhibited higher abundance in captive tigers (Figure 4A). According to the LEfse result of the enzymes, many families had significantly higher in wild tigers, including glycoside hydrolases (GH) families, carbohydrate esterases (CEs), and carbohydrate-binding modules (CBMs), conversely, in captive tigers, the main difference in GH families (Figure 4B).

### Relationship Between the Gut Microbiota Compositions and Functions

To identify the patterns of covariation, we clustered the taxonomy ID from wild individuals into three clusters by using the ward method. Cluster 1 was dominated by Firmicutes (*Clostridium*), Cluster 2 was dominated by Firmicutes (*Enterococcus* and *Paeniclostridium*), and the remaining taxonomy bacteria belong to Cluster 3, which contains five phyla and are mainly composed of Firmicutes (*Blautia*), Actinobacteria (*Actinoplanes*), and Proteobacteria (*Campylobacter*). According to the results of the correlation analysis, we found that Cluster 2 had no relationship with function groups and Cluster 1 had promoting function on transport and catabolism, cell motility, endocrine system, and infectious disease of bacterial. Cluster 3 had a significant positive correlation with most of the functional groups.

The clustering pattern of gut microbiota of captive Amur tiger was similar to that of wild tiger. Cluster 1 was Firmicutes (*Blautia*) and the abundance of *Blautia* in this group accounts for 59.95% of the total content of *Blautia* in captive Amur tigers, Cluster 2 includes Firmicutes (*Clostridium* and *Blautia*) and Proteobacteria (*Escherichia*), and the remaining taxonomy bacteria belong to Cluster 3, which contains five phyla and are mainly composed of Bacteroidetes (*Bacteroides*), Firmicutes (*Clostridium*), and Actinobacteria (*Dietzia*). Nevertheless, all taxonomy ID clusters had a significant relationship with different functional groups. Cluster1 had significant positive correlation with membrane transport and aging and immune system, while Cluster 3 had an inhibitory relationship with the function of substance dependence. The correlations between pathway partition and taxonomy ID clusters were compared to analyze the functional differences of gut microbiota between wild and captive tigers. We found that taxonomy ID of wild tigers were positive associated with the categories of metabolism (lipid metabolism, biosynthesis of other secondary metabolites, xenobiotics biodegradation, and metabolism), environmental information processing (membrane transport and signal transduction), cellular processes (transport and catabolism and cell motility), and organismal systems (endocrine system). While the taxonomy ID of captive tigers had significant relationship with functions of digestive system, substance dependence, and infectious disease of parasitic.

## Discussion

Gut microbiota, which is closely related to the host health, play an important role in impacting the body’s metabolism, immunity, speciation, and many other functions (Bravo et al., 2011, 2012; Brucker and Bordenstein, 2012; Ezenwa et al., 2012). Analysis of the differences in gut microbiota is a key step in releasing captive Amur tigers to help expand the wild population. While, due to technology and samples collection of wild tigers, this part of the research is still limited. In this study, we performed a metagenomic inventory of 22 wild individuals and 13 captive individuals to investigate the variations of the gut microbiota composition and function traits and their correlations both in wild and captive Amur tigers. Taxonomic assignment of 16S rRNA sequences showed that the gut microbiota of Amur tigers was composed by 17 bacterial phyla, and the most important constituents were Firmicutes and Proteobacteria and this is in agreement with previous study on composition and functional structures of captive tigers (He et al., 2018b), gaur (Prabhu et al., 2020), and other mammals (Wasimuddin et al., 2017). At the genus level, *Collinsella* was dominant and positively correlated with circulating insulin. It was suggested that the abundance of *Collinsella* depends on the dietary intake of the host (Gomez-Arango et al., 2018), which significantly more abundance in captive tigers.

### Gut Microbiota Structures and Composition of Amur Tigers

The alpha diversity showed significant difference, captive tigers show a higher gut microbial diversity, which contrasts our predictions and may be caused by the reasons that captive tigers get more chances to contact with other individuals and interact with their keepers and visitors more often. The high microbial richness of captive population was also reported in leopard seals (Nelson et al., 2013). Moreover, we found that wild tigers harbor higher difference in unweighted UniFrac distance, which implies that the composition of the gut microbiota among wild individuals showed less overlap than captive ones and have higher divergence of gut microbiota when compared with captive ones. According to the result of PCoA and Anosim, there was a clear difference between these two groups, demonstrating that the bacterial communities differed greatly between wild and captive populations.

As shown in Figure 2D, the differences at genus levels between captive and wild tigers were obvious. The relative abundance of *Sphingomonas* and *Lysinibacillus* were remarkably higher in the wild tigers, while *Blautia*, *Fusobacterium*, and *Bacteroides* had a significant increase in captive tigers. *Sphingomonas* and *Lysinibacillus* had a widespread distribution in various aquatic environments and contaminated soils and sediments (Failor et al., 2019) and were characterized by the functions of degrading the copper pipes in drinking water and strong enzymatic capabilities, respectively (White et al., 1996; Welch, 2006; Xu et al., 2017; Failor et al., 2019). The increased relative abundance of *Blautia* and *Bacteroides* was also found in human when the body had a good nutrition (Durand et al., 2017) or had consumed high protein and fat food (Lee et al., 2019). Bacteria of phylum of Actinobacteria, Fusobacteria, and Bacteroidetes were significantly more prevalent in captive tigers and these phyla were involved in maintaining homeostasis of the host, the potential for protein degradation, and responsibility of the body’s metabolism (Colston and Jackson, 2016). The reasons for the composition differences in the gut microbiota of wild and captive Amur tigers may be due to the combined effect of diet and habitat heterogeneity. Previous studies have also proved that these two factors greatly contribute to gut microbial variation within species (Amato et al., 2013; Barelli et al., 2015; Uddin et al., 2017). Wild Amur tiger has a wide range for activities, as well as a variety of vegetation types and rich food resources. There are more than 10 kinds of animals that were preyed by tigers, and wild tigers often successfully hunt large prey about once a week (Gu et al., 2018). However, captive tigers were fed by duck, pork, and beef as their common dietary items (He et al., 2018a). Regularly, continuous feeding and less exercise were typical common characteristics of captive animals. Therefore, in the gut microbiota of wild Amur tigers, a variety of gut taxonomy are commonly found in the environment, and the dominant bacteria in captive tigers are related to nutrients consumption.

### Amur Tiger Core Microbiota

For the Amur tiger core microbiome, we identified three phyla as the main core microbiome, which have been confirmed by previous report about the human core microbiome (Zaura et al., 2009; Zhu et al., 2010). According to the results of *t* test, *Faecalimonas*, *Lachnoclostridium*, and *Blautia* were significantly increased in captive tigers. *Lachnoclostridium* abundance has been shown to increase in the gut microbiome of pigs after feeding the low-protein diet supplemented with 10 g/kg of alpha-ketoglutarate (Zhou et al., 2020). *Faecalimonas umbilicata*, as the only type species of *Faecalimonas* genus, had the function of acetate-producing bacterium in human feces (Mitsuo et al., 2017; Sakamoto et al., 2018). All positive associations among three main phyla control the entire network and are consistent with the generally self-sustaining assortment of bacteria (Aschenbrenner et al., 2017). Firmicutes, as the most prevalent phylum in the core microbiota and the largest modules in the bacterial network, could breakdown carbohydrates and promote the absorption of nutrients (Colston and Jackson, 2016). We also identified the positive correlation between Firmicutes and Actinobacteria members, which may indicate their consistent response to similar environmental conditions (Eiler et al., 2011). All of these results suggested that diet was the main reason for the differences in the core taxa of Amur tigers.

### Differences in Gut Microbiota Functions Between Wild and Captive Amur Tigers

This study uncovered the significant differences in biological functions of the gut microbiota between wild and captive Amur tigers. Twelve pathways belonging to five major categories (environmental information processing, cellular processes, organismal systems, human diseases, and not included in pathway or brite) were more abundant in wild tigers. A large part of these functions was related to human diseases that have essentially influenced by environmental factors (Kanehisa et al., 2009) or organism systems and played an important role in ensuring the normal function of the host and maintaining a stable state (Xing et al., 2019). These pathways are more prevalent in wild tigers, possibly because they faced more diverse conditions in the wild than in captive tigers, such as expanding their territory and home range and hunting various prey to obtain energy supply. One of the two functions, which were more significant in captive tigers, belongs to the metabolism that helps the host to digest and absorb (Dai et al., 2011). This situation could be explained by the stable food intake, and relatively little exercise consumes energy in captive tigers.

LEfSE analysis based on the CAZy databases showed that the largest proportion differences between the two populations are GH families. The relative abundance of GH2, GH3, GH16, GH20, GH29, GH35, GH89, GH92, GH97, and GH109 were higher in captive tigers, whereas GH17, GH24, and GH73 were higher in wild tigers. GHs had a crucial role in breakdown complex carbohydrates (Lee et al., 2013) and played an indispensable role in processing various exogenous and endogenous glycoconjugate in human gut microbiota (Pellock et al., 2018). This result was consistent with the hypothesis that captive Amur tigers in stable food sources required more enzymes for metabolism than wild tigers (Pitta et al., 2016), proving that disturbing effects of dietary interventions.

### Relationship Between the Gut Microbiota Compositions and Functions

We tried to confirm the relationship between gut microbiota and metabolism function; however, it was somewhat unrealistic to treat each taxonomy ID and each function as independent (Hooper et al., 2002) while the taxonomy ID clusters and classifications of KEGG Level2 were closely correlated to each other. By the correlation result, we found that the Firmicutes (*Clostridium*) in wild tigers was positively correlated with the function of infectious disease, a similar conclusion was reached from another analysis showed that *Clostridium perfringens* was the second most common bacteria that cause bacterial illnesses (Scharff, 2012; Huang et al., 2019). According to our results, Firmicutes (*Blautia*) was positively correlated with the immune system. Previous reports from Jenq et al. (2015) also indicate that *Blautia* had a beneficial anti-inflammatory effect. Most of the correlation results of this study showed that multiple gut microbiota work together on the functions. The only significant inhibitory effect was recognized between Cluster 3 and substance dependence (alcoholism, cocaine addiction, and amphetamine addiction) function; this phenomenon may be related to some intake of medications for disease treatment and prevention for captive tigers, which would also alter the gut microbiota community considerably (Xue et al., 2013; Qiao and Ma, 2018). The Cluster 3 of taxonomy ID in wild tigers could promote the three main categories of environmental information processing, cellular processes, and organismal systems, which were also the main differences of gut microbiota functions between wild and captive tigers. In addition, we found that Firmicutes (*Clostridium* and *Blautia*) and Proteobacteria (*Escherichia*) were associated with parasitic infectious diseases. *C. perfringens* belongs to the genus of *Clostridum*, and its spores had been identified in water in previous studies. Managers may be needed to supervise the quality of drinking water to reduce the presence of pathogen bacteria and achieve the purpose of inhibiting the prevalence of function.

### Research Implications

Since differences in gut microbiota between captive and wild tigers were largely influenced by dietary habits and living environment (Xue et al., 2013; Bletz et al., 2016; Schmidt et al., 2019), we suggest that adapting the gut microbial community of captive tigers to that of wild tigers should be considered as one of the important preparation stages for reintroduction and an indispensable metric to evaluate whether reintroduction should be attempted. Furthermore, to achieve this, we propose the following considerations to be used in the reintroduction preparation process:

Rewilding training should not only consider an animal’s predation ability but also its adaptability to food and environment. Additionally, the rewilding site should be located in a natural environment similar to where the tiger will be introduced. Over time, their diet should be gradually replaced by accurate potential prey from the wild, and we should minimize human and drug intervention. Ideally, the rewilding training site would gradually increase in size over time until it is large enough to include most of the wild potential prey species and habitat types in the site to help the tigers establish a wild-adapted gut microbiota.

It is also necessary to extend the duration of rewilding training to make sure that gut microbiota could successfully evolve from captive structure to a wild structure. A monitoring database on the gut microbiota of wild Amur tigers and their dynamics should be established as contrasting data. Once the rewilding training is able to significantly reduce the difference in composition and function of gut microbiota between captive and wild tigers, the reintroduction will likely have a higher chance of success.

Further research needs to shed more light on understanding the interactions among host genetic relatedness, environmental variation, dietary changes, and the gut microbiota of Amur tigers. Additionally, incorporating novel methods (e.g., transcriptome) to study the functional annotation of gene content and the functional traits of hosts should be implemented to better understand the impact that physiology and immunology of tigers may further impact their reintroduction success.

## Conclusion

Our study provided a comprehensive catalog of the gut microbiome of Amur tigers through 16S rRNA gene and metagenome analysis of fecal samples. Comparing analysis identified significant variations of gut microbiota composition and functions between captive and wild populations and also indicated that diet and environment have a great influence on these variations. These findings were of great significance for the reintroduction of captive Amur tigers, given that the differences of gut microbiota composition and functions between captive and wild tigers would greatly impact the ability of captive tigers to adapt to the wild environment. For further study, incorporating novel methods (e.g., transcriptome) to study the functional annotation of gene content and the functional traits of host would be essential for better understanding the physiology and immunology of tigers.

## Data Availability Statement

The datasets generated for this study can be found in the raw sequences of 16S rRNA gene that were available in the NCBI Sequence Read Archive under BioProject PRJNA576784 with the accession number SRP225682. The gut metagenome sequencing was deposited under the BioProject PRJNA633309 with the accession number SRP262119.

## Author Contributions

GJ, JM, and JQ conceived and designed this study. YN and GJ wrote the manuscript and carried out field and laboratory work. JQ, MD, and SC provided suggestions and polished the manuscript. YN, XL, JW, and JQ made the figures. All authors contributed to the article and approved the submitted version.

## Conflict of Interest

The authors declare that the research was conducted in the absence of any commercial or financial relationships that could be construed as a potential conflict of interest.

## Footnotes

^1^http://www.chinadaily.com.cn/global/2019-07/30/content_37496613.htm

^2^http://bowtie-bio.sourceforge.net
